# Hundreds of rice bodies in the subacromial-subdeltoid bursa: report of two cases and literature review

**DOI:** 10.1186/s12891-020-03563-0

**Published:** 2020-08-12

**Authors:** Jiong Jiong Guo, Kailun Wu, Yingjie Xu, Huilin Yang

**Affiliations:** 1 Department of Orthopedics, The First Affiliated Hospital of Soochow University, 188 Shizi St, Suzhou, 215006 China; 2 Department of Orthopedics, Suzhou Dushuhu Public Hospital (The Affiliated Dushuhu Hospital of Soochow University), Suzhou, China

**Keywords:** Shoulder, Subacromial-subdeltoid bursa, Rice bodies, Arthroscopy, Chopsticks technique

## Abstract

**Background:**

Multiple rice bodies (RB) in the shoulder joint is a rare disorder of unknown etiology that requires percutaneous drainage or surgical operation.

**Case presentation:**

We reported arthroscopic removal of hundreds of RB in the subacromial-subdeltoid bursa in two cases by our “chopsticks technique”. One was associated with seropositive rheumatoid arthritis and the other was a rare synovial origin possibly due to microinfarction and ischemia after the radiotherapy. Radical debridement of necrotic tissue, “red tissue” and synovitis by arthroscopic radiofrequency ablation was essential for eliminating the cause of RB. A favorable clinical evolution was observed for both patients.

**Conclusions:**

We highlight the importance of patient-specific differential diagnosis and the clinical course of RB to help us further understand the pathogenesis of this uncommon disorder. Meanwhile, evacuation of RB and “red tissue” ablation by arthroscopy showed good results.

## Background

Rice bodies (RB) were initially reported as intra-articular proteinaceous masses associated with tuberculosis infection [1]. After that there were sparse reports of RB, rice body formation is associated with infectious arthritis (tuberculosis, atypical mycobacterial infection), rheumatoid arthritis, traumatic arthritis, seronegative arthritis, juvenile arthritis, osteoarthritis and chronic bursitis [2–4]. However, where most reports attentions have been placed on investigating the etiology, radiological appearances and different rare sites, little has been performed on the quantity, clinical course and treatment details of RB [2, 3, 5]. We present two cases of arthroscopic removal of hundreds of RB in the subacromial-subdeltoid bursa by chopsticks technique with more than a two-year follow-up. We also counted the definite number of bodies. These results add to our current knowledge about RB in the shoulder.

## Case presentation

### Case 1

A 27-year-old woman presented with the complaint of a painless mass in her right shoulder that had been growing over the last 4 months. She had a history of rheumatoid arthritis for 5 years. Physical examination revealed a cystic swelling over the anterior aspect of the right shoulder with fusiform shape. Her preoperative active range of flexion was 160°, abduction was 100°, external rotation with the elbow at the side was 70°, and her hand could be placed on the back to the level of the 12th thoracic vertebra. Erythrocyte sedimentation rate (41 mm/hr) and C-reactive protein levels (6.36 mg/dL) were elevated. Evaluation for rheumatoid arthritis revealed an elevation in antibodies to cyclic citrullinated peptides (80.45 RU/mL). Magnetic resonance imaging (MRI) suggested a markedly distended subacromial/subdeltoid bursa, which was full of multiple nodules varing in size. These nudules were isointense on T1 and T2 weighted spin-echo sequences. The rotator cuff was intact and the glenohumeral joint appeared no articular cartilage injury or wear.

**Fig. 1:**
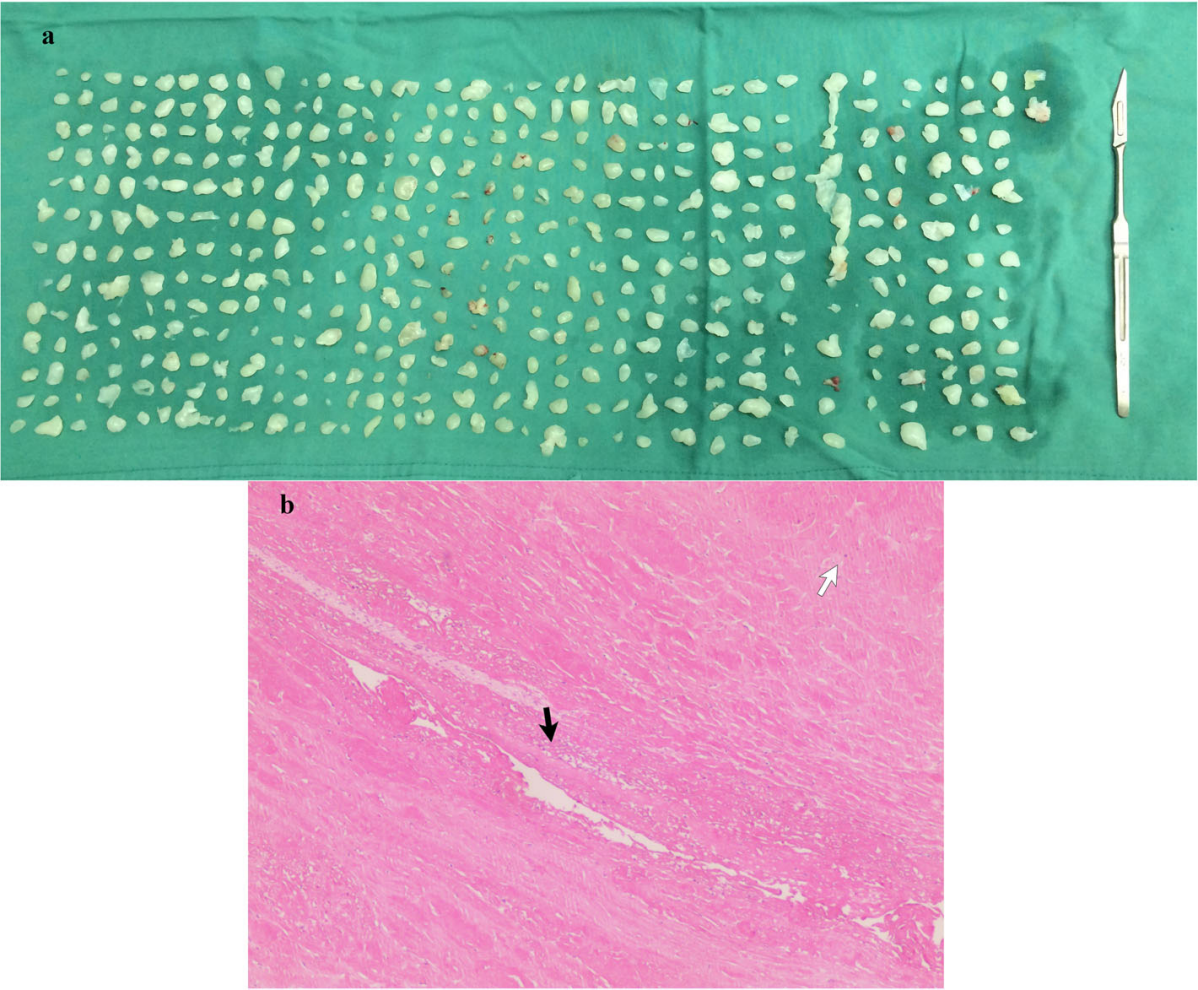
Gross and pathological photograph in Case 1. **a** Gross photograph showed 525 spherical rice bodies with the range of 2.5–18 mm in diameter. **b** Rice bodies was composed of a collagenous core with chronic inflammatory cells and necrosis (white arrow) surrounded by fibrin layer (black arrow) (H&E, × 100)

Arthroscopic shoulder surgery was performed after careful preparation. The patient was placed in the beach-chair position with the arm forward flexed with 3 kg of skin traction applied, secured his neck and body, and the back of the chair was at an angle of approximately 60 degrees to the floor [6]. Before the shoulder is placed in traction, and before it is wrapped by a compression self-adhering elastic bandage, it is important to adequately pad the hand and forearm with gauze or foam in order to avoid injuries to the skin from traction and to avoid excessive nerve compression about the forearm and wrist. The setup optimizes arthroscopic visualization by distracting the humerus from the acromion so that the potential space is maximized. The standard posterior “soft spot” portal was used to visualize the subacromial space. Intraoperatively, hundreds of grain-like loose bodies were found to fill the subacromial and subdeltoid bursae. The bodies were picked up with straight forceps and removed one by one. The inflamed bursa and other tissues were ablated by Coblator (ArthoCare Corporation, Sunnyvale, CA). We defined these tissues as “red tissue” because of red color under the scope. Five hundred twenty-five bodies were collected finally ranging in size from 2.5 mm to 18 mm. (Fig. 1a) The pathological findings demonstrated chronic active inflammation with fibrin deposition and necrosis formation, but no granulomas. RB was mainly composed of fibrous tissue wrapping necrosis and a mass of chronic inflammatory cells with a prominent granulomatous response. Histochemical staining for acid fast bacilli was negative. (Fig. 1b) The patient recovered uneventfully at a two-year follow-up. This was in accord with a diagnosis of rheumatoid-arthritis-associated subacromial-subdeltoid bursitis with rice body formation.

### Case 2

**Fig. 2:**
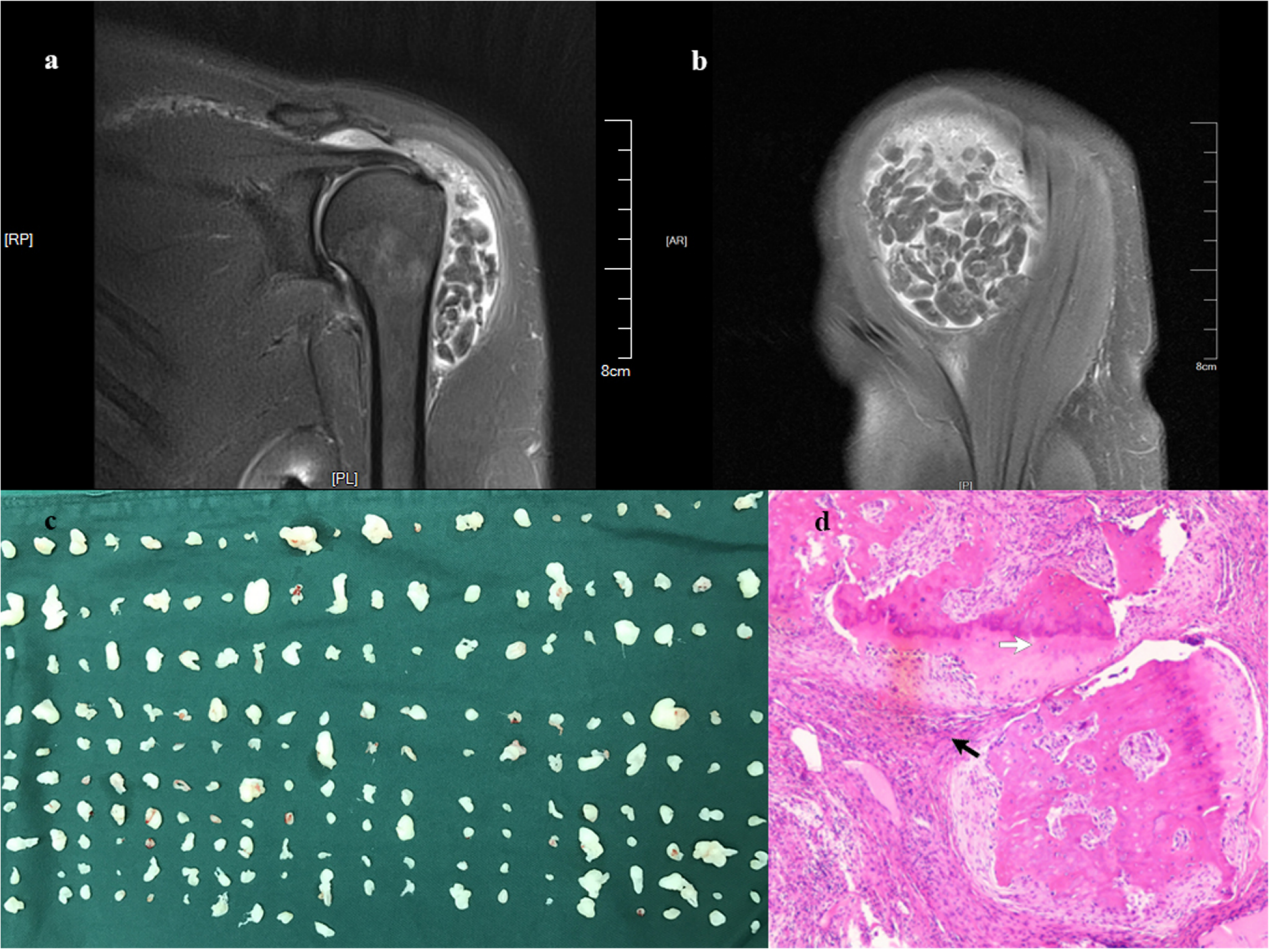
MRI, specimens, and pathological findings in Case 2. **a**, **b** Coronal fat-suppressed T1-weighted coronal and axial images after gadolinium enhancement clearly demonstrated multiple tiny rice bodies within the subacromial-subdeltoid bursa, and subcoracoid bursa. **c** Intra-operative photograph demonstrated 313 numerous shiny rice body masses with different sizes. **d** Photomicrograph showed infltration of chronic inflammatory cells (black arrow) with fbrin deposition, numerous osteochondral structure (white arrow) (H&E, × 200)

A 45-year-old woman, with a diagnosis of breast cancer, was referred by her oncologist for an orthopaedic review of her left shoulder swelling for 5 months. She received mastectomy 6 years ago and radiotherapy after that. She had no medical history of rheumatoid arthritis, tuberculosis or previous allergies. Her active range of motion was restricted. External rotation with the elbow at the side was 50°. Forward elevation was 130° and abduction was 80°. Erythrocyte sedimentation rate and C-reactive protein levels were normal. Antibodies to cyclic citrullinated peptides was 14.34 RU/mL, a little high than normal (0.00–5.00 RU/mL). RF was positive. MRI of the left shoulder showed numerous tiny nodules in the subacromial and subdeltoid bursa, which were surrounded by a moderate degree of bursa fluid. The particles were isointense on both T1- and T2- weighted images, without enhancement after intravenous Gadolinium. (Fig. 2a, b) Rheumatologist did not give her any medication for rheumatologic condition and thought more about the radiotherapy.

Arthroscopic surgery was performed under the beach-chair position. Three hundred thirteen grain-like nodules filled the subacromial and subdeltoid bursa. (Fig. 2c, d) All the rice body masses were removed by “chopsticks” technique with straight forceps. Because RB characteristics are soft and smooth, straight forcep is the ideal instrument to pick up them. “Red tissue” was ablated by Coblator as much as possible. She had improved at 3 months follow-up review with an increases in abduction of 40° (80° to 120°), an increase of 10° (50° to 60°) in external rotation with the elbow at the side, and by three vertebrae in the height at which they could place their hand on their back. The patient received no medication subsequently and there was no recurrence after 2 years.

## Literature review process and results

An English literature search was conducted on PubMed, MEDLINE and EMBASE covering the span from 2004 to 2015. Search items included rice body, shoulder, arthroscopy, treatments. Articles without treatment and outcome were excluded. We focused on reviewing studies of symptom, surgical procedures, pathogenesis and outcomes.

After screening of all abstracts, four articles were included. One study reported two elderly cases with short-time recurrence after synovectomy and removal of rice bodies [7]. Furthermore, there were two rare cases. One was an infantile case with polyarticular RB, the other was a rice body formation associated with bioabsorbable suture anchor after arthroscopic repair [8, 9]. Subramaniam et al. reported the treatment of giant RB caused by rheumatoid arthritis [4]. In our series, we described chopsticks technique to remove pathogenic factor, which achieved favorable effects. The results of these reports were summarized in Table 1.

**Table 1:** Selected literature review of rice bodies in shoulder for comparison of outcomes reported

Authors/ Published Year	Age/ sex	Location	Symptom	Procedures	Pathogenesis	Follow-up time	Outcome
Tan et al./ 2004 [7]	70/M	Right shoulder	S, full ROM	Synovectomy arthroscopic drainage and removal of rice bodies	Chronic synovitis and synovial chondromatosis	6 months	Recurrence and Re-operation
	88/F	Left shoulder	S, P and decreased ROM	Ditto	Arthritis and synovial chondromatosis	4 months	Recurrence, pain but good ROM
Mutlu et al./2004 [8]	4/F	Left shoulder and knee	S, decreased ROM	Removal of rice bodies but not synovium	Unknown	1-year	No recurrence
Subramaniam et al./2012 [4]	49/F	Right shoulder	S, P and decreased ROM	Removal of giant rice bodies, subacromial and subdeltoid bursitis	Rheumatoid Arthritis	30 months	No recurrence
Sivaloganathan et al. /2015 [9]	60/M	Left shoulder	S, P and decreased ROM	Debridement, removal of rice bodies, anchor and suture removal.	Post-operation of shoulder (poly-L-lactic acid anchor)	2-year	No recurrence
Guo et al. (current study)	27/F	Right shoulder	S, full ROM	Chopsticks technique and removal of “red tissue”	Rheumatoid arthritis	2-year	No recurrence
	45/F	Left shoulder	S, decreased ROM	Ditto	Radiotherapy	2-year	No recurrence

*S* swelling, *P* pain, *ROM* range of movement

## Discussion and conclusions

We presented two cases of RB in the shoulder with arthroscopic debridement and evacuation of all loose bodies. Careful arthroscopic exploration of the subacromial, subdeltoid and subcoracoid bursae to reveal all bodies was a hard job. The proliferative nature of the inflamed synovium can obscure the arthroscopic view. Loose bodies of synovial origin and eburnation of articular cartilage to bone (especially of the humeral head) were noted during the shoulder arthroscopic procedure. Consequently, we could not count the definite number of bodies by arthroscopic drainage. Generally, many surgeons used the shaver to remove and put these multiple loose bodies into the tray. But crushing of bodies because of shaver was common. In order to add our knowledge of RB, we removed and counted the definite numbers of bodies in the subacromial and subdeltoid bursa. Beach-chair position with the arm held in flexion optimizes arthroscopic visualization by distracting the humerus from the acromion [6]. Although requiring arthroscopic skills and patience, our technique seems to be safe and reproducible, and it provides significant functional improvements in the patients with RB, also with aesthetic benefits in young female patients.

The cause of rice body formation remains obscure, but is most likely an unusual complication of chronic bursitis. However, seldom was there any underlying pathology reported. The first case in the study was associated with seropositive rheumatoid arthritis. Some investigators have suggested microinfarctions after intra-articular synovial inflammation and ischaemia, with subsequent synovial shedding and encasement by fibrin derived from synovial fluid as a possible cause [10, 11]. Based on the results of previous studies in vivo of local temporary ischemia on the effectiveness of ionizing radiation-based anti-cancer therapy, we thought an alteration of the synovial fluid after radiotherapy is also a possible mechanism of the second case. A severe inflammatory bursitis with RB formation related to the poly-L-lactic acid anchor was reported [9]. Clinical characteristics, radiologic and laboratory findings of RB have been reported. To our knowledge, the definite numbers and best treatment of RB have not been reported. Calabozo et al. reported arthroscopic drainage with intra-articular urokinase for synovial effusions with rice bodies [12]. Ergun et al. mentioned open surgical intervention of RB in the thickened common flexor tendon sheath of the wrist [13]. Lui reported a patient with idiopathic tenosynovitis and RB formation at the anterior ankle [5]. It was treated with tendoscopic synovectomy and was complicated by pseudoaneurysm of the dorsalis pedis artery.

RB formation is usually associated with bursitis, inflamed synovial shedding, or other rheumatoid soft tissue changes. Another advantage of arthroscopy is Coblator, which can ablate those inflamed tissue easily and completely. Radical debridement of the inflamed bursa and its contents was important for the prognosis. There was neither communication with the underlying glenohumeral joint nor obvious compromise of the rotator cuff. These “red tissues” are different from pannus, which is a sheet of inflammatory granulation tissue that spreads from the synovial membrane and invades the glenohumeral joint in rheumatoid arthritis. Our findings were consistent with previous reports noting that necrotic tissue, “red tissue” or synovitis played a vital role in the development of the formation of rice body [4, 7–9].

In conclusion, we describe two cases of RB in female patients, one had rheumatoid arthritis and the other had a history of breast cancer. Beach-chair positioned shoulder arthroscopy was performed combined with bodies removal, counting, and debridement of inflamed soft tissue. We highlight the significance of arthroscopic chopsticks technique and debridement of “red tissue” response to inflammation as much by ablation. Further studies are necessary to better understand the relation among the surgical, radiological findings and prognosis.


Additional file 1

## Data Availability

All data concerning the case are presented in the manuscript.

## Abbreviations

RB: Rice bodies
CA: Coblator
MRI: Magnetic resonance imaging
